# Classification, History, and Future Prospects of Maxillofacial Prosthesis

**DOI:** 10.1155/2019/8657619

**Published:** 2019-07-18

**Authors:** Fernanda Pereira de Caxias, Daniela Micheline dos Santos, Lisiane Cristina Bannwart, Clovis Lamartine de Moraes Melo Neto, Marcelo Coelho Goiato

**Affiliations:** ^1^Department of Dental Materials and Prosthodontics, São Paulo State University (UNESP), School of Dentistry, Araçatuba, São Paulo, Brazil; ^2^Center for Oral Oncology, UNESP, Araçatuba, São Paulo, Brazil

## Abstract

This review presents a classification system for maxillofacial prostheses, while explaining its types. It also aims to describe their origin and development, currently available materials, and techniques, predicts the future requirements, and subsequently discusses its avenues for improvement as a restorative modality. A literature search of the PubMed/Medline database was performed. Articles that discussed the history, types, materials, fabrication techniques, clinical implications, and future expectations related to maxillofacial prostheses and reconstruction were included. Fifty-nine articles were included in this review. Maxillofacial prostheses were classified as restorative or complementary with subclassifications based on the prostheses finality. The origin of maxillofacial prostheses is unclear; however, fabrication techniques and materials have undergone several changes throughout history. Currently, silicones and acrylic resins are the most commonly used materials to fabricate customized prostheses. Maxillofacial prostheses not only restore several types of orofacial defects but also improve the patients' quality of life. Although the current clinical scenario concerning the field of maxillofacial prostheses is promising, improvements in material quality and techniques for maxillofacial prostheses may be expected in the future, to produce better results in the treatment of patients.

## 1. Introduction

Maxillofacial deformities are embarrassing to patients and may negatively affect their physical and psychological health, potentially resulting in serious psychiatric, familial, and social problems [1]. These deformities can be congenital, caused by malformation and developmental disturbances, or acquired, caused by pathologies such as necrotizing diseases and oncosurgeries or trauma [2].

Plastic (or autoplastic) surgery is generally preferred over alloplastic (or artificial) reconstruction, when appropriate [3, 4]. Nevertheless, several congenital and acquired defects still require prosthetic restoration [3].

In 1953, Ackerman defined maxillofacial prostheses as the phase of dentistry that repairs and artificially replaces parts of the face after injuries or surgical intervention [5]. This definition excluded the use of prostheses to treat congenital craniofacial deformities in an effort to improve facial aesthetics [6]. Maxillofacial reconstruction involves implanting artificial substitutes for intraoral and extraoral structures such as the eyes, ears, nose, maxilla, mandible, esophagus, cranial bones, and palate [7]. Maxillofacial prostheses are primarily fabricated using acrylic resin and/or silicone [8], according to the facial structure of the patient. The prostheses are retained and supported by a number of structures such as osseointegrated implants [9], the remaining skin with or without adhesive [10], body cavities [11], and teeth [12].

Maxillofacial prostheses have an important impact on the patient's quality of life and self-esteem, as they can immediately correct the defects that occur after surgical procedures [5]. The prostheses allow individuals to reintegrate into their social and familial environments, making them happier and more confident. In order to achieve success, it is necessary to integrate different health professionals, such as doctors, nurses, psychologists, physiotherapist, speech therapists, and dentists for prosthetic rehabilitation.

Several materials, techniques, and clinical approaches have been used for maxillofacial prostheses. This review presents a classification system for maxillofacial prostheses, explains the different types of prostheses, describes their origin and evolution, identifies current materials and techniques, predicts future needs, and discusses improvements for this restorative modality.

## 2. Materials and Methods

A literature search of the PubMed/Medline database was conducted using the keywords “maxillofacial prosthesis” and “maxillofacial prosthesis AND history.” Articles that discussed the history, types, materials, fabrication techniques, clinical implications, and future expectations related to maxillofacial prostheses and reconstruction were included. The search was widened, as necessary, and references cited in the publications were also included as part of this study. There was no limit regarding the year of publication or language of articles. Fifty-nine articles were included in the present review.

### 2.1. Classification of Maxillofacial Prostheses

**Figure 1:**
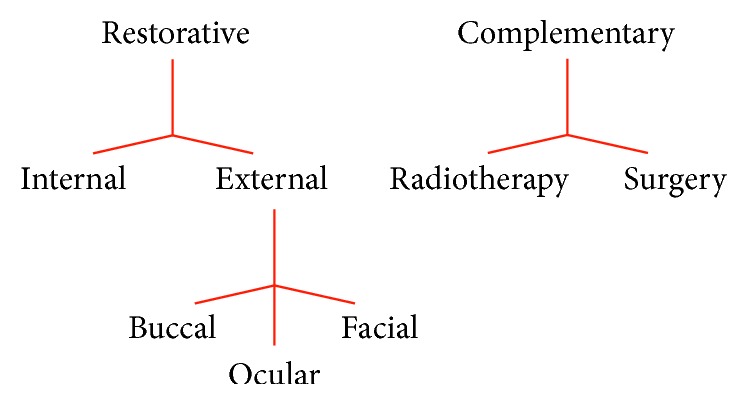
Representative scheme of the classification of maxillofacial prostheses.

In general, maxillofacial prostheses can be classified as restorative or complementary. Restorative prostheses substitute for bone loss or repair deformities of facial contour. They can be located internally within the tissue or externally as oral, ocular, or facial prostheses. Complementary prostheses help with plastic surgery, in the pre-, trans-, or postoperative period, or in radiotherapy sessions (Figure 1).

#### 2.1.1. External Buccal Prostheses

The aim of prosthetic treatment for intraoral damage is to restore the patient's masticatory function and phonetics, improve aesthetics, and reestablish their psychosocial well-being [3]. When intraoral reconstructive surgery is contraindicated, palatal obturator [3], mandibular [13, 14], and tongue prostheses can be used for the treatment [15–17].

#### 2.1.2. Palatal Obturator Prostheses

**Figure 2:**
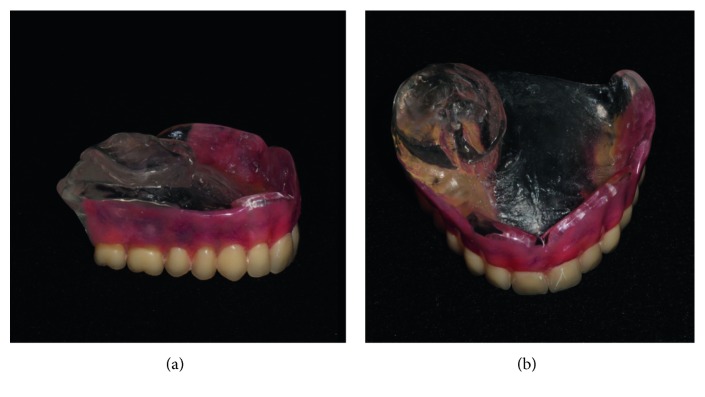
Palatal obturator prosthesis.

Patients with uni- or bilateral defects may have facial collapse, difficulty with mastication and swallowing, unintelligible speech, and lower quality of life [18]. Palatal obturator prostheses (Figure 2) are fabricated to close the communication between the oral and nasal cavities, restoring speech and improving chewing and swallowing of the patient [18].

Patients who have undergone maxillectomy may demonstrate poor support for the prosthesis, thus possibly impairing its stability and retention capability. According to Wang, the factors that affect the prognosis for a prosthesis are size of the defect, number of remaining teeth, quantity of healthy tissue, quality of the mucosa, exposure to radiotherapy, and the patient's ability to accept the prosthetic treatment [19].

The stability and retention of an obturator prosthesis depends on different factors such as size and location of the defect, number of remaining teeth, and the support area of the remaining palate [20]; that is, the larger the defect, the fewer the remaining teeth, and the smaller the support area, the worse the stability and retention [20].

Total maxillary resection has an unfavorable prognosis [20]. In such cases, a multidisciplinary team is essential to develop and implement an adequate treatment plan [21] that preserves the healthy structures and, when possible, applies bone or skin grafts on the sinus cavity wall [20].

The obturator prostheses can be fabricated before the surgery and applied immediately after it to protect the surgical cavity. Alternatively, it can be temporary, fabricated few weeks after the surgery allowing time for customization and tissue repair. Restorative, or definitive, prostheses are fabricated after healing. They have all the characteristics of conventional prostheses, are more functional, and result in better aesthetics [22].

#### 2.1.3. Mandibular Prostheses

Partial or total mandibulectomy impairs the whole stomatognathic system. Therefore, surgery and prosthetic reconstruction are particularly difficult [23]. The larger the resection, the worse the prognosis for the patients to retain dentition [13]. The tumor dimension and location, the extension of the tongue, the degree of soft tissue involvement, and the number of remaining teeth after a mandibulectomy are important factors that influence the success of restorative treatments [14].

Regardless of the amount of tissue removed from the mandible, the surgery causes several functional and aesthetic sequelae for the patient [14, 23]. The consequences include decrease in masticatory quality [14, 23, 24] and impact on facial appearance, speech impairment, malocclusion, swallowing difficulties, worsened quality of life [14, 23], and xerostomia caused by radiotherapy [14].

Mucosupported complete dentures or removable partial prostheses may only partially restore lost aesthetic qualities. However, the function remains impaired since the treatment cannot be optimized due to articular changes and a shortened prosthetic basal area.

#### 2.1.4. Tongue Prostheses

Carcinomas commonly affect the lateroposterior surface of the tongue, and the treatment often involves surgical excision and radiotherapy [15, 16]. In cases of large lesions, surgical resection can include the mouth floor and the tongue [15, 16]. Mastication and swallowing can be impaired, causing liquid and food accumulation in the oral cavity [16, 17], and patients have unintelligible speech [15, 16]. Furthermore, the tongue removal results in instability of mandibular prostheses in edentulous patients [17].

The fabrication of an artificial tongue with a posterior inclination, to guide the alimentary bolus to the oropharynx, and an anterior elevation, for articulation of dentilingual phonemes and vowels [16], improves the patients' ability to chew, swallow, and speak. In addition, use of the palatography technique [25] eliminates sibilant distortions, improving intelligibility of speech [15]. It is prudent to refer the patient to a speech therapist before, during, and after the treatment to improve his/her speech and to increase the tone of surrounding muscles to assist with oral functions.

#### 2.1.5. Ocular Prostheses

Partial or total eye loss not only results in vision loss but also impacts the patient's self-esteem and social life [1]. A primary purpose of ocular prostheses is to allow for reintegration into the society since the eyes are an important factor in human relations [1].

Furthermore, the ocular prostheses also function to retain tone of the upper eyelid muscles, preserve the tear duct to avoid eyelash adherence and conjunctival dryness, prevent eyelid atresia due to lack of function, and protect the cavity mucosa from debris and dust [26]. Ocular bulb loss results from pathologic or accidental causes. Three types of orbit and eyelid surgeries are related to ocular prostheses: evisceration, the partial removal of the eye bulb while preserving the sclera; enucleation, the complete removal of eye bulb with only the capsule and oculomotor muscles remaining; and exenteration, the removal of all contents of the orbital cavity and surrounding tissues [2].

A well-adapted prosthesis requires simple maintenance. The patient removes it daily for cleaning [27, 28] with water and neutral soap [28]. The efforts necessary for the techniques involved in the fabrication of eye prostheses aim to assist the patients who need it in the numerous complex aspects associated with the loss of vision and organ mutilation.

#### 2.1.6. Facial Prostheses

In general, facial prostheses can be classified as nasal, lip, oculopalpebral, auricular, skullcap, and traqueostomal. There are also prostheses for large facial reconstructions. These prostheses artificially reconstruct soft and hard tissues which were previously lost [29], to restore the patient's appearance, leading to improved self-esteem and quality of life [30].

Although facial prostheses primarily function to restore aesthetics, they also have other physiological functions. For example, the nasal prosthesis improves airflow and speech [31]. Lip prostheses seal the lips and reestablish lip support, to ensure better chewing, swallowing, and speech [32]. Auricular prosthesis improves hearing in noisy environments. Skullcap prostheses protect the brain [33]. Traqueostomal prostheses allow breathing, speech [34], and filtering the air. According to Neves and Vilela, an aesthetically pleasant facial prosthesis must mimic and reproduce the lost shape, volume, position, texture, translucency, and color [35], in order to make sure that the prostheses are almost imperceptible to an observer [35].

#### 2.1.7. Radiotherapy Prostheses

Radiotherapy prostheses are an alternative treatment for patients with malignant tumors [36]. These prostheses, also known as radium-holder apparatus, allow radioactive elements to be oriented to treat the tumor, attenuating the doses absorbed by adjacent healthy tissues [36]. They are used for brachytherapy or external actinotherapy by contact [36].

These prostheses can be made with resin or silicone, and their fabrication involves a team of well-integrated specialists: a radiotherapist, a physicist, and a prosthetic dentist [36]. After the dentist fixes the catheters, the computer plans the correct distribution of therapeutic doses to each tumor area [36].

### 2.2. History of Maxillofacial Prostheses

#### 2.2.1. Past

The origin of maxillofacial prostheses is not clear [37]. According to Conroy, earliest known application of engineering principles to restore facial appearance and dental occlusion may be attributed to Hippocrates [38]. The Etruscans society was considered to be advanced in the art of intraoral prosthesis with remains of prosthetic structures found in their ancient burial sites [37]. Mummified Egyptians were found with enamel-covered, silver eyes with bronze lids [39] as well as nasal and auricular structures [38]. However, this does not mean that these prostheses were used during the people's lives [38, 39]. Nevertheless, there is evidence that Romans used the artificial eyes *in vivo*. Similar to the Egyptians, the ancient Greeks fabricated artificial eyes with silver and placed them in their statues [39]. Bulbulian cited the work of Popp (1939) who described the use of artificial eyes and noses by the Chinese and Indians in ancient times [37]. Evidence of oculofacial prostheses in China (year 200 AD) suggests that their designs were based on a metal framework externally coated with a layer of lacquer to simulate facial skin tones [38]. A motivation for the use of these prostheses in Roman, Egyptian, and Indian societies could possibly be the amputation of ears, noses, and hands as a punishment for crimes [37, 38]. The only recorded case of maxillofacial reconstruction between 200 AD and 1000 AD is related to the Byzantine Emperor Justinian II who had a golden nose manufactured while he was incarcerated [38].

Ambroise Paré provided the first documented use of maxillofacial prostheses during the 16th century [37, 38]. This French surgeon mentioned the use of the artificial eyes [37, 39], ears, and noses and described the manufacturing of an obturator prosthesis [37]. The prostheses idealized by Paré were made with different materials like papier-mâché, leather, ivory, gold, and silver [38]. Nasal prostheses could be retained by sticky substances or by three linen strips wrapped around the patient's head. Ear prostheses could be retained by a metal band that was placed over the patient's head. The ocular prostheses could be retained internally in the orbit or externally retained similar to the ear prostheses [37, 38]. Regarding the obturator prostheses, a dry sponge could be attached to the upper surface of the prosthesis (obturator region) so that when the dry sponge entered the palatine cavity and was moistened, it expanded and held the prosthesis in position [37].

Glass and wood were also used to fabricate maxillofacial prostheses as they became more common in Europe during the 16th century. Doctors from Germany and France debated which material was better for manufacturing prostheses [38]. Later, during the 17th century, Pierre Fauchaud recognized that maxillofacial prostheses could not only improve mastication but also repair palatal defects and improve aesthetics [37]. Fauchaud designed a palatal obturator with wings that were folded together during insertion into the palatal defect. Once positioned, the wings spread out to hold the prosthesis in place [37]. Fauchaud also improved the aesthetics of artificial teeth. The ivory artificial teeth were covered with a thin metal layer, and again this metal layer was covered with enamel [37]. In 1681, artificial eyes were made of enamel aiming to “look natural” [39].

In the 19th century, William Morton used a gold plate to fabricate an obturator prosthesis. Morton constructed an artificial nose of porcelain, which was attached to the patient's glasses [37]. In the same century, some prostheses were made of nitrated cellulose (discovered in 1867). However, for smokers, nitrated cellulose produced unsatisfactory results, as it turned brown and caught fire [38]. Later, in France, cellulose acetate was used with better clinical results. Celluloid was also used for cranioplasty [38].

At the end of the 19th century, vulcanite was introduced for the fabrication of maxillofacial prosthesis [37]. This material replaced the celullose, metals, ceramics, and other materials used for the manufacture of prostheses at the time. Additionally, vulcanite was frequently used during World War I (1914–1918) to manufacture maxillofacial prostheses [37]. Some prosthodontists used other materials, such as a thermoplastic material based on wax reinforced with resin and a material based on gelatin and glycerin [38]. Despite delivering satisfactory results when new, the gelatin and glycerin prostheses only lasted few days or, at most, a week [37].

Despite ocular prostheses demand during World War I, when more than 600,000 soldiers had head and facial injuries, government regulations hampered the manufacture of glass ocular prostheses [38, 39]. Maxillofacial prosthetics had an important role in the quality of life for recuperating soldiers since they were able to engage in social activities and go out in public [38].

For much of the first half of the 20th century, maxillofacial prosthetics were related to the reconstruction of the cleft palate. An important exception was in army and navy hospitals [7]. Between World Wars I and II, researchers tried to develop better materials for the fabrication of maxillofacial prostheses, such as prevulcanized latex [38]. An increase in the number of injured people due to the war led to the creation of specialized plastic and maxillofacial surgery units in the United Kingdom and British Colonies [38]. The first of these units was utilized in 1939 [38]. The new materials developed after this era are still in use.

#### 2.2.2. Present

Currently, the materials used to fabricate maxillofacial prostheses include vinyl plastisol, acrylic resins based on polymethyl methacrylate (PMMA), polyurethanes, latex, and silicone polymers [40, 41]. Silicones and acrylic resins are the most used materials for maxillofacial reconstruction [8, 28, 41].

The material of choice for fabrication of facial prostheses is silicone polymers that are classified as one of two types: room temperature vulcanizing silicone and high-temperature vulcanizing silicone [41]. Silicone polymers have several advantages, including chemical inertness, strength, durability, and ease of manipulation [40]. Two major disadvantages of silicone polymers are color degradation and instability, caused by exposure to ultraviolet rays, air pollution, temperature variation, and humidity [42]. Silicones are widely used but still need improvement because they last for short periods, such as 6 months, and need frequent replacement [43].

The acrylic resins have been used to fabricate intraoral prostheses, such as obturators and ocular prostheses [41]. It can be thermopolymerized (by water bath or microwave energy) or autopolymerized [44]. Da Silva tested the biocompatibility of different polymerization methods on a human conjunctival cell line and concluded that heat polymerization using a water bath was the most appropriated method to fabricate ocular prostheses [44]. With the advent of acrylic resins, ocular prostheses have become much more versatile, resistant, and comfortable to use. They can be shaped and adapted to irregularities in the anophthalmic cavity producing a more accurate, safer (the materials are inert and nontoxic), and practical final cosmetic result [28]. Moreover, orbital implants (i.e., hydroxyapatite, polyethylene, aluminum oxide, and silicone) can also be used to restore orbital volume and some mobility for the prosthesis [28].

**Figure 3:**
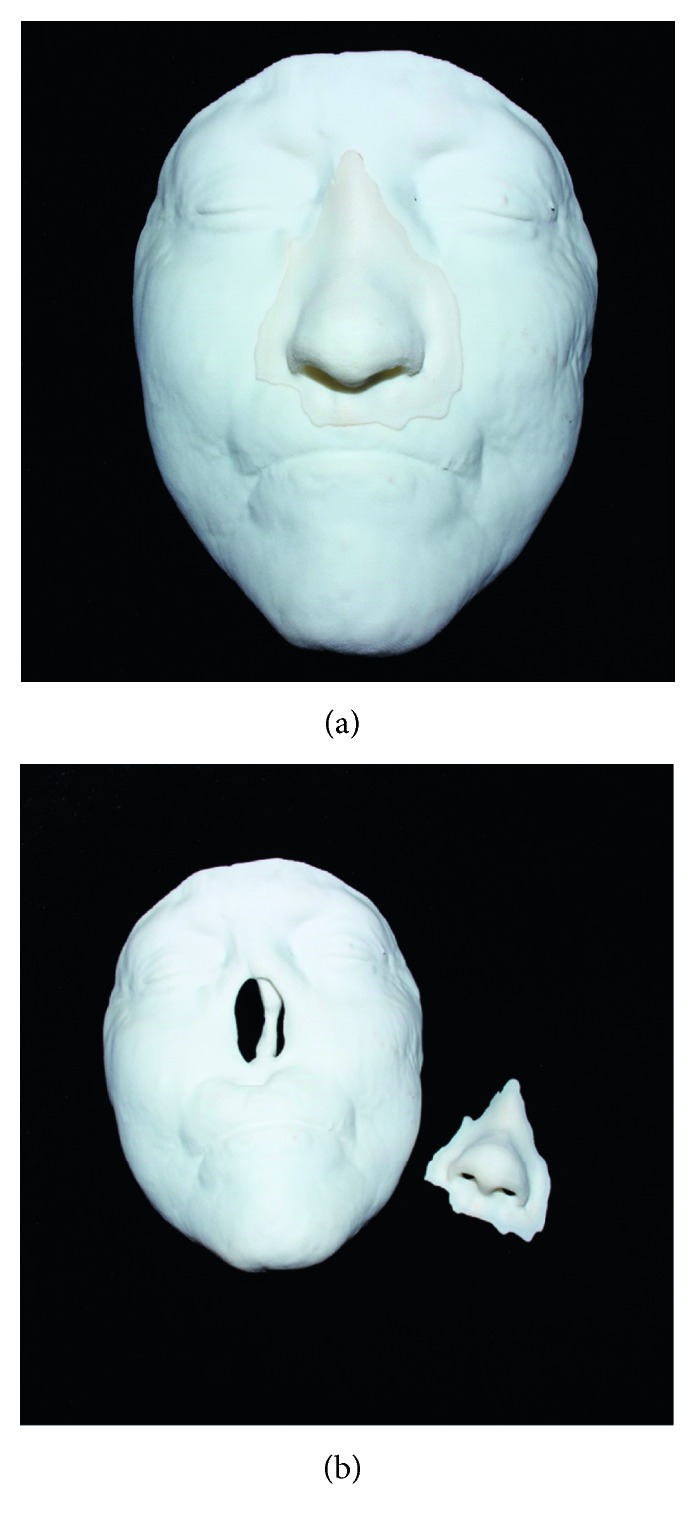
Prototyping of a nose prosthesis.

Different methods can be used to hold the external prostheses in place, depending on the area and type of defect. They can be held in a cavity mechanically; placed on the skin using adhesives; supported with osseointegrated implants, which have been used for maxillofacial rehabilitation since 1979 [8]; or retained by magnets [13]. Three-dimensional (3D) printing is a new, evolving technology that has the potential to revolutionize medical education and maxillofacial reconstruction (Figure 3) [45]. It allows the creation of customized, patient-specific models to optimize facial reconstruction by providing anatomical precision and individualized solutions for facial reconstruction [45]. 3D printing can make prosthetic treatments more accurate, faster, and less expensive [45]. Silicone rubber can also be infused with colored pigments in order to print prostheses that match the patient's skin color [45].

The future of maxillofacial prosthetics depends on the development of new materials and techniques, as well as changing clinical expectations regarding head and neck defects.

#### 2.2.3. Future

A large number of studies point to the development of new materials and techniques to optimize the treatment of congenital and acquired orofacial defects. Recent studies identified several areas for further investigation when evaluating different properties of maxillofacial prostheses and their management, such as biocompatibility [46], cleaning protocols [47], pigment incorporation [48], and material bonding efficiency [49]. Ferreira [14] foresaw the development of new prostheses that substitute for bone tissue without requiring bone grafts, thus reducing the morbidity and the recovering time, as a possible future approach in maxillofacial reconstruction. According to Ferreira [14], these new prostheses should be produced using engineering, computer-aided design and manufacturing (CAD-CAM), and surgical guides [14].

Several steps in the fabrication of maxillofacial prostheses are still artisanal, requiring time and skill [28]. Modern techniques for ocular prosthesis fabrication, such as 3D printing and digital imaging, are able to reduce the treatment time, better replicate the patient characteristics [28, 45], eliminate taking facial impressions, and reduce the complexity of wax pattern sculpting [50]. However, modern techniques still need improvements, along with reduced cost and wider availability, to lead to a promising future for maxillofacial reconstructions.

In addition to technical advances, the expectations related to the future for maxillofacial prostheses will be determined by the needs of patients. The world population of individuals aged 60 years or over was 962 million in 2017 [51]. The United Nations estimates that there will be 2.1 billion older people by 2050 worldwide [51]. Aging is linked to deteriorating health and an increased risk of cancer [52]. Then, there may be an increase in cases of head and neck cancers and an increased demand for maxillofacial prosthetics and reconstructions over the next few decades. Military medicine will continue to play an important role in facial reconstructions to treat lost function and improve damaged appearances of war victims [50]. It is important to consider the fact that Middle East and Africa are regions in constant war, and there will probably be more conflicts in this region in the future [53]. This fact highlights the importance of maxillofacial prosthetic rehabilitation for the war victims. Besides wars, other episodes of violence lead to facial disfiguration. For example, acid attacks are generally targeted at the head and neck area causing eye perforations [54], nasal deformities, microstomia, skin deformation, and other consequences [55].

## 3. Conclusion

Maxillofacial prostheses restore several types of orofacial defects as well as improve the patient's quality of life. This is an ancient treatment modality that has developed over centuries. The current situation is promising, and there are positive expectations for the future.
